# Supplementary material for: The Effects of Twitter Sentiment on Stock Price Returns
Source: PLoS One. 2015 Sep 21;10(9):e0138441. doi: 10.1371/journal.pone.0138441 (PMC4577113; doi:10.1371/journal.pone.0138441)
Supplement: S1 Appendix — Detailed information about the detected events from the Twitter data and their polarity. We show the 118 detected EA events and 182 detected non-EA events. (PDF) [file pone.0138441.s001.pdf]

S1 Appendix

The Effects of Twitter Sentiment on Stock Price Returns

This part contains detailed information about the detected events from the Twitter data. In particular, we here show:

- the 118 detected EA events (Table 1),
- the 182 detected non-EA events (Table 2).

Table 1: The 118 EA events detected, and their polarity.

Ticker	Date (d)	Event polarity	P_d	Ticker	Date (d)	Event polarity	P_d
AXP	2013-07-17	Neu	0.239	KO	2013-07-16	Neg	-0.349
AXP	2013-10-16	Pos	0.903	KO	2013-10-15	Neu	0.323
AXP	2014-04-16	Neg	0.129	KO	2014-04-15	Neu	0.229
BA	2013-10-23	Pos	0.700	KO	2014-07-22	Neg	0.019
BA	2014-01-29	Neg	0.059	MCD	2013-07-22	Neg	-0.186
BA	2014-04-23	Pos	0.758	MCD	2013-10-21	Neg	-0.195
BA	2014-07-23	Neu	0.382	MCD	2014-01-23	Neg	-0.206
CAT	2013-07-24	Neg	-0.594	MCD	2014-04-22	Neg	-0.552
CAT	2013-10-23	Neg	-0.521	MCD	2014-07-22	Neg	-0.356
CAT	2014-01-27	Neu	0.425	MMM	2013-07-25	Pos	0.812
CAT	2014-07-24	Neg	0.047	MMM	2013-10-24	Pos	0.910
CSCO	2013-08-14	Neg	-0.052	MMM	2014-01-30	Neg	0.017
CSCO	2014-02-12	Neu	0.201	MMM	2014-04-24	Neg	-0.104
CSCO	2014-05-14	Neu	0.482	MMM	2014-07-24	Pos	0.957
CSCO	2014-08-13	Neu	0.226	MRK	2013-07-30	Neg	0.028
CVX	2013-08-02	Neg	0.041	MRK	2014-02-05	Neu	0.612
CVX	2013-11-01	Neg	-0.309	MRK	2014-07-29	Pos	0.888
CVX	2014-01-31	Neg	-0.595	MSFT	2013-10-24	Neu	0.590
CVX	2014-05-02	Neg	0.007	MSFT	2014-04-24	Neu	0.697
CVX	2014-08-01	Neu	0.456	NKE	2013-06-27	Neu	0.516
DD	2013-07-23	Pos	0.714	NKE	2013-12-19	Pos	0.875
DD	2013-10-22	Pos	0.757	NKE	2014-03-20	Neu	0.487
DD	2014-01-28	Pos	0.847	PFE	2013-07-30	Neu	0.179
DD	2014-04-17	Neg	-0.285	PFE	2013-10-29	Neu	0.478
DD	2014-07-22	Neu	0.619	PFE	2014-01-28	Neu	0.614
DIS	2013-08-06	Neu	0.477	PG	2013-08-01	Pos	0.744
DIS	2013-11-07	Pos	0.746	PG	2013-10-25	Pos	0.825
DIS	2014-02-05	Pos	0.782	PG	2014-01-24	Neu	0.405
DIS	2014-05-06	Pos	0.722	PG	2014-04-23	Neu	0.413
GE	2013-07-19	Neu	0.488	PG	2014-08-01	Neu	0.418
GE	2013-10-18	Neu	0.688	T	2013-07-23	Neu	0.554
GE	2014-01-17	Neu	0.418	T	2013-10-23	Neu	0.459
GE	2014-04-17	Neu	0.688	TRV	2013-07-23	Neu	0.617
GE	2014-07-18	Pos	0.811	TRV	2013-10-22	Pos	0.842
GS	2013-07-16	Neu	0.280	TRV	2014-01-21	Neg	-0.088
GS	2013-10-17	Neg	-0.262	TRV	2014-04-22	Pos	0.942
GS	2014-01-16	Neg	-0.067	TRV	2014-07-22	Neg	-0.302
GS	2014-07-15	Neu	0.603	UNH	2013-07-18	Pos	0.907
HD	2013-08-20	Pos	0.778	UNH	2013-10-17	Neg	-0.333
HD	2013-11-19	Pos	0.810	UNH	2014-01-16	Neu	0.365
HD	2014-02-25	Neu	0.638	UNH	2014-04-17	Neg	-0.923
HD	2014-05-20	Neg	-0.077	UNH	2014-07-17	Neu	0.672
HD	2014-08-19	Pos	0.919	UTX	2013-07-23	Pos	0.763
IBM	2013-07-17	Neg	0.098	UTX	2014-01-22	Neu	0.589
IBM	2014-01-21	Neg	-0.232	UTX	2014-04-22	Pos	0.813
IBM	2014-04-16	Neg	-0.313	UTX	2014-07-22	Neu	0.465
INTC	2013-07-17	Neg	-0.380	V	2014-01-30	Neu	0.689
INTC	2013-10-15	Neu	0.538	VZ	2013-07-18	Pos	0.878
INTC	2014-01-16	Neg	0.102	VZ	2013-10-17	Neu	0.600
INTC	2014-04-15	Neu	0.564	VZ	2014-01-21	Neu	0.693
JNJ	2013-07-16	Pos	0.880	VZ	2014-04-24	Neg	-0.041
JNJ	2013-10-15	Pos	0.711	VZ	2014-07-22	Pos	0.926
JNJ	2014-01-21	Neu	0.538	WMT	2013-08-15	Neg	-0.667
JNJ	2014-04-15	Pos	0.807	WMT	2014-02-20	Neg	-0.753
JNJ	2014-07-15	Pos	0.777	WMT	2014-08-14	Neg	-0.387
JPM	2013-07-12	Pos	0.789	XOM	2013-08-01	Neg	-0.188
JPM	2013-10-11	Neg	-0.436	XOM	2014-01-30	Neg	-0.387
JPM	2014-01-14	Neg	-0.102	XOM	2014-05-01	Neu	0.209
JPM	2014-04-11	Neg	-0.673				
JPM	2014-07-15	Neu	0.380				

Table 2: The 182 non-EA events detected, and their polarity.

Ticker	Date (d)	Event polarity	P_d	Ticker	Date (d)	Event polarity	P_d	Ticker	Date (d)	Event polarity	P_d
AXP	2013-06-20	Neg	-0.333	INTC	2013-08-19	Neu	0.638	PFE	2013-06-12	Neg	-0.517
AXP	2013-07-31	Neg	-0.047	INTC	2013-11-22	Neg	-0.150	PFE	2013-12-17	Neg	0.043
AXP	2013-09-10	Pos	1.000	INTC	2014-06-13	Pos	0.748	PFE	2014-03-12	Neg	-0.565
AXP	2013-11-14	Pos	1.000	JNJ	2013-06-17	Neu	0.649	PFE	2014-04-28	Pos	0.714
AXP	2013-12-24	Neg	-0.348	JNJ	2013-09-06	Neu	0.393	PFE	2014-05-19	Neg	-0.551
AXP	2014-05-27	Pos	0.794	JNJ	2013-11-04	Neg	-0.869	PFE	2014-06-24	Pos	0.991
AXP	2014-07-24	Neu	0.466	JNJ	2014-01-16	Pos	0.925	PFE	2014-07-21	Pos	0.991
BA	2013-07-12	Neg	-0.159	JPM	2013-07-30	Neg	-0.971	PG	2013-08-05	Pos	1.000
BA	2013-09-17	Pos	0.796	JPM	2013-09-19	Neg	-0.852	PG	2013-08-30	Neu	0.259
BA	2013-11-18	Pos	0.776	JPM	2013-10-21	Neg	-0.754	PG	2013-10-08	Pos	0.750
BA	2014-03-10	Neg	-0.460	JPM	2013-11-19	Neg	-0.736	PG	2013-11-14	Pos	0.914
BA	2014-06-11	Neg	-0.628	JPM	2014-01-07	Neg	-0.790	PG	2014-04-09	Pos	0.745
BA	2014-07-18	Neg	0.057	JPM	2014-03-07	Neg	0.100	PG	2014-06-12	Neu	0.459
BA	2014-09-08	Pos	0.928	JPM	2014-04-14	Neg	0.006	PG	2014-07-11	Neg	-0.400
CAT	2013-07-17	Neg	-0.736	KO	2013-09-30	Neu	0.153	PG	2014-08-29	Neg	-0.444
CAT	2013-09-20	Neg	-0.444	KO	2014-02-06	Neu	0.650	T	2013-07-12	Pos	0.873
CAT	2013-10-18	Pos	0.755	KO	2014-04-23	Neg	-0.297	T	2013-12-06	Neu	0.600
CAT	2013-12-06	Pos	0.826	KO	2014-06-17	Neu	0.617	T	2014-05-19	Neg	0.032
CAT	2014-04-01	Neg	-0.024	KO	2014-08-14	Pos	0.858	T	2014-07-30	Neu	0.478
CAT	2014-05-22	Neu	0.255	MCD	2013-06-10	Neu	0.312	TRV	2014-08-28	Neg	-0.793
CAT	2014-07-29	Pos	0.718	MCD	2013-07-17	Neg	-0.657	UNH	2013-07-02	Neg	-0.090
CSCO	2013-07-23	Pos	0.915	MCD	2013-08-27	Neu	0.652	UNH	2013-09-10	Neu	0.666
CSCO	2013-11-15	Neg	0.077	MCD	2013-09-27	Neu	0.454	UNH	2013-10-21	Neg	-0.300
CSCO	2014-08-15	Neg	-0.596	MCD	2013-11-15	Neg	-0.157	UNH	2014-01-07	Pos	0.859
CVX	2013-08-28	Pos	0.862	MCD	2013-12-09	Neg	-0.630	UNH	2014-06-04	Pos	1.000
CVX	2014-03-11	Pos	0.733	MCD	2014-01-09	Neu	0.268	UNH	2014-08-27	Pos	0.809
CVX	2014-05-28	Pos	0.753	MCD	2014-02-17	Pos	0.983	UTX	2013-06-18	Neg	0.090
DD	2013-06-14	Neg	-0.333	MCD	2014-03-10	Neg	-0.637	UTX	2013-10-02	Neg	-0.895
DD	2013-07-17	Pos	0.777	MCD	2014-04-04	Neg	-0.187	UTX	2013-12-13	Neg	-0.272
DD	2013-10-24	Pos	0.875	MCD	2014-05-22	Neg	-0.685	UTX	2014-01-27	Pos	0.793
DD	2013-11-22	Pos	1.000	MCD	2014-08-08	Neg	-0.708	UTX	2014-05-21	Pos	1.000
DD	2014-01-09	Neu	0.642	MMM	2013-06-10	Pos	1.000	UTX	2014-07-24	Neu	0.250
DD	2014-05-15	Neu	0.440	MMM	2013-08-23	Pos	1.000	V	2013-07-31	Neu	0.164
DD	2014-06-27	Neg	-0.407	MMM	2013-12-17	Pos	0.806	V	2013-09-10	Neg	-0.389
DD	2014-07-29	Pos	0.853	MMM	2014-08-28	Neu	0.166	V	2013-12-11	Pos	0.755
DIS	2013-07-02	Pos	0.903	MRK	2013-06-14	Neu	0.384	V	2014-01-17	Neu	0.652
DIS	2013-09-12	Pos	0.877	MRK	2013-08-20	Neu	0.666	V	2014-03-21	Neu	0.389
DIS	2013-10-22	Pos	0.971	MRK	2013-10-16	Pos	0.750	V	2014-05-28	Pos	0.750
DIS	2013-12-23	Neu	0.659	MRK	2014-01-13	Pos	0.888	VZ	2013-06-26	Neu	0.517
DIS	2014-09-01	Pos	0.818	MRK	2014-04-10	Pos	0.957	VZ	2013-08-29	Pos	0.805
GE	2013-06-18	Pos	0.937	MRK	2014-05-07	Neg	0.073	VZ	2014-02-24	Neu	0.342
GE	2013-07-09	Neu	0.583	MRK	2014-06-09	Pos	0.729	VZ	2014-05-16	Pos	0.826
GE	2013-08-06	Neu	0.473	MRK	2014-08-13	Neg	0.019	VZ	2014-07-29	Neu	0.529
GE	2013-08-30	Neg	0.076	MRK	2014-09-04	Pos	0.964	WMT	2013-07-12	Pos	0.886
GE	2014-09-01	Pos	1.000	MSFT	2013-07-11	Pos	0.756	WMT	2013-08-29	Neu	0.454
GS	2013-09-10	Neg	-0.315	MSFT	2013-09-03	Neg	0.063	WMT	2013-09-25	Neg	-0.760
GS	2014-02-11	Neu	0.303	MSFT	2013-11-06	Pos	0.836	WMT	2013-11-25	Neu	0.402
GS	2014-04-02	Neg	-0.264	MSFT	2014-02-04	Pos	0.802	WMT	2014-01-02	Neg	-0.889
HD	2013-06-26	Neu	0.600	MSFT	2014-03-18	Pos	0.884	WMT	2014-01-31	Neg	-0.693
HD	2013-08-14	Neu	0.272	MSFT	2014-05-20	Neu	0.283	WMT	2014-03-18	Neu	0.321
HD	2013-12-11	Pos	0.891	MSFT	2014-06-23	Pos	0.966	WMT	2014-04-16	Neg	-0.405
HD	2014-02-12	Pos	0.767	MSFT	2014-07-17	Neu	0.391	WMT	2014-05-07	Neu	0.193
HD	2014-07-10	Neg	-0.352	NKE	2013-07-01	Neu	0.481	WMT	2014-06-23	Pos	0.938
HD	2014-09-02	Neg	-0.295	NKE	2013-08-01	Pos	0.800	WMT	2014-07-24	Neg	-0.333
IBM	2013-07-09	Neg	-0.518	NKE	2013-09-10	Neg	-0.350	XOM	2013-08-19	Neg	-0.122
IBM	2013-08-06	Neg	-0.650	NKE	2013-10-09	Pos	0.903	XOM	2013-09-27	Pos	0.833
IBM	2013-10-29	Pos	0.835	NKE	2013-11-21	Pos	1.000	XOM	2013-11-14	Pos	0.960
IBM	2014-02-10	Pos	0.714	NKE	2014-01-30	Neu	0.654	XOM	2013-12-16	Pos	0.838
IBM	2014-06-04	Pos	0.720	NKE	2014-05-21	Pos	0.986	XOM	2014-03-19	Neu	0.466
IBM	2014-07-03	Pos	0.988	NKE	2014-06-23	Pos	0.989	XOM	2014-09-03	Neu	0.266
IBM	2014-09-03	Neu	0.157	NKE	2014-09-05	Pos	0.957				
